# LD_50 _values of ISS or NISS from the Chinese Trauma Data Bank and their application in predicting post-trauma sepsis

**DOI:** 10.1186/cc12961

**Published:** 2013-11-05

**Authors:** Huaping Liang, He Jin, Zheng Liu, Ya Xiao

**Affiliations:** 1 Department 1, Research Institute of Surgery, Daping Hospital, The Third Military Medical University, Chongqing, People's Republic of China

## Background

To introduce the LD_50 _values of ISS or NISS from the Chinese Trauma Data Bank, and to compare the roles of some formulae including LD_50 _values in predicting the onset of post-trauma sepsis.

## Materials and methods

Data of 6,542 patients (aged ≥16) from the Chinese Trauma Data Bank were analyzed to obtain the LD_50 _values of ISS or NISS using probit regression. A total of 908 trauma patients (aged ≥16) admitted to Daping Hospital of Chongqing from January 2011 until June 2013 were enrolled, Discrimination and calibration of the ISS, NISS, SIRS score (the first 3 days post trauma), ISS/LD_50ISS_, NISS/LD_50NISS_, ISS´SIRS score, NISS´SIRS score, ISS/LD_50ISS_´SIRS score, NISS/LD_50NISS_´SIRS score, ISS+SIRS score, NISS+SIRS score, ISS/LD_50ISS_+SIRS score, and NISS/LD_50NISS_+SIRS score in predicting post-trauma sepsis were compared using receiver operator characteristic (ROC) curves and Hosmer-Lemeshow statistics.

## Results

The LD_50 _values of ISS for age 16 to 44, 45 to 64, and ≥65 were 55, 49, and 33, respectively (for males: 54, 50, and 31; for females: 60, 47, and 39). The LD_50 _values of NISS for age 16 to 44, 45 to 64, and ≥65 were 62, 56 and 43, respectively (for males: 62, 56, and 42; for females: 65, 53, and 48). The predicting capability of ISS/LD_50ISS_+SIRS score and NISS/LD_50NISS_+SIRS score were equivalent (area under the ROC curve = 0.932 versus 0.932) and both showed better discrimination than others in predicting post-trauma sepsis. For ISS/LD_50ISS_+SIRS score, the cutoff value of ROC curve was 2.2128, with a positive predictive value of 65.86%, a negative predictive value of 95.95%, a sensitivity of 87.13%, a specificity of 87.11%, a positive likelihood ratio of 6.76, a negative likelihood ratio of 0.15, a Youden index of 0.7424, and an accuracy of 87.11%. For NISS/LD_50NISS_+SIRS score, the cutoff value of ROC curve was 2.3208, with a positive predictive value of 66.87%, a negative predictive value of 95.98%, a sensitivity of 87.13%, a specificity of 87.68%, a positive likelihood ratio of 7.07, a negative likelihood ratio of 0.15, a Youden index of 0.7481, and an accuracy of 87.56%.

## Conclusions

This study calculates for the first time the LD_50 _values of ISS or NISS from Chinese trauma patients. The novel and simple formulae ISS/LD_50ISS_+SIRS score and NISS/LD_50NISS_+SIRS score are then set up to predict the incidence of sepsis following traumatic injury, which perform better at predicting capability than ISS, NISS, SIRS score and other formulae including LD_50 _values of ISS or NISS.

